# An integrated approach of community health worker support for HIV/AIDS and TB care in Angónia district, Mozambique

**DOI:** 10.1186/1472-698X-9-13

**Published:** 2009-07-17

**Authors:** Sandrine Simon, Kathryn Chu, Marthe Frieden, Baltazar Candrinho, Nathan Ford, Helen Schneider, Marc Biot

**Affiliations:** 1 Médecins Sans Frontières, Angónia, Mozambique; 2 Médecins Sans Frontières, Johannesburg, South Africa; 3 Department of Health, Angónia, Mozambique; 4 Infectious Diseases Epidemiology Unity, University of Cape Town, Cape Town, South Africa; 5 Médecins Sans Frontières, Maputo, Mozambique

## Abstract

**Background:**

The need to scale up treatment for HIV/AIDS has led to a revival in community health workers to help alleviate the health human resource crisis in sub-Saharan Africa. Community health workers have been employed in Mozambique since the 1970s, performing disparate and fragmented activities, with mixed results.

**Methods:**

A participant-observer description of the evolution of community health worker support to the health services in Angónia district, Mozambique.

**Results:**

An integrated community health team approach, established jointly by the Ministry of Health and Médecins Sans Frontières in 2007, has improved accountability, relevance, and geographical access for basic health services.

**Conclusion:**

The community health team has several advantages over 'disease-specific' community health worker approaches in terms of accountability, acceptability, and expanded access to care.

## Introduction

Sub-Saharan Africa faces a dire shortage of human resources for health, a situation exacerbated by the overwhelming demands of the dual epidemics of HIV/AIDS and tuberculosis (TB) that have swept over the region [1]. Community health workers (CHWs) were promoted as part of the Alma Ata Declaration thirty years ago in order to increase access to basic health care services and to reach "health for all [...] by the year 2000." Subsequently, different types of CHW were engaged to support primary care services, but there was considerable variation in their background, role, motivation, and the quality of services they provided. Despite some successful experiments, enthusiasm for CHW programmes declined in subsequent decades, mainly due to difficulties in recreating successful models at the national level [2,3].

In resource-limited settings, the CHW approach has regained credibility in the last few years through its support of HIV/AIDS care, in particular voluntary testing and counselling (VCT), and treatment adherence support for people on HIV and TB treatment [4]. Community engagement is also gaining support in some specific disease areas such as for the diagnosis and treatment of malaria [5] and maternal-child health [3]. These vertical initiatives have however been criticized as contributing to fragmented service delivery and heterogeneous coverage [3].

This paper provides a participant-observer perspective of the evolution of CHWs from vertical and isolated activities for TB, HIV and other specific diseases to an integrated community health team approach for tackling the main disease burden in a rural district of Mozambique.

### Setting

Mozambique ranks 172 out 177 countries in the UNDP Human Development Index [6]. In this context of low basic human development, access to health care is limited by a lack of qualified health workers with only three medical doctors per 100,000 inhabitants – seven times less than the minimum standard set by the World Health Organization [7]. Moreover, more than half the country's medical doctors work in the capital, Maputo [8]. In Angónia, there is only one medical doctor per 112,000 inhabitants. In this context, the burden of HIV and TB is placing a substantial additional burden on the health system. HIV prevalence in Mozambique is estimated at 14–17% among the adult population [9], while the TB incidence rate is 460 per 100,000 inhabitants per year with a TB mortality rate of 129 per 100,000 inhabitants per year. The HIV/TB co-infection rate is around 48% [10]. TB treatment coverage is limited: in 2007 only 41% (38,044) of TB patients were registered on TB treatment by the national TB programme.

Angónia district in Tete Province (northwestern Mozambique) is a rural district with a population of approximately 336,000 inhabitants spread over 3,437 km^2 ^(population density 98 inhabitants per km^2^) [11]. This under-resourced district benefits from only one rural hospital, eight health centres and one health post; many of these health facilities are located along the main roads, leaving large areas without any proximal health services. Public transport is very unreliable, and during the five-month long rainy season (from November to March) some areas are virtually inaccessible due to overflowing rivers and waterlogged roads.

The diseases causing the highest morbidity and mortality in Angónia district are malaria, respiratory infections, malnutrition and diarrhoea [12]. TB and the growing HIV epidemic are also increasingly significant public health problems. The case detection rate for TB is low, with only 8.4% of the approximately 1,500 expected cases [13] diagnosed and treated in 2007. As an indication of HIV burden, 7.8% of all VCT clients tested positive, while 50% of TB patients are HIV co-infected [14].

VCT is provided in nearly all health centres, however turnaround is limited by a shortage of nurses and laboratory capacity. Antiretroviral therapy (ART) is available in only two health centres in the district, resulting in a significant treatment gap: as of June 2008, 735 people were receiving ART, leaving an estimated 2,000 people in need [14].

While nationwide a provincial TB-HIV and malaria coordinator is responsible for translating policy into an effective comprehensive provincial response, the scarcity of the human resources at district level is paralysing many efforts.

### Evolution of community support

The Ministry of Health (MoH) of Mozambique introduced the concept of the lay CHW through the "Agente Polivalente Elementar" in the 1970s to support public health activities at the district level [15]. Various types of CHW engagement have been piloted in Angónia district over the years and many are still active now. The recent increase in TB and HIV activities has added additional layers of community engagement. These are described below.

In 2003 the MoH, together with the international medical organisation Médecins Sans Frontières (MSF), launched a project to address the problem of HIV/AIDS in the district by providing information and education to the community, access to counselling and testing, and treatment of opportunistic infections. In 2005, in order to decrease the hospitalization requirement for TB treatment (the first two months of treatment) and increase case detection of TB (the main opportunistic infection of people living with HIV/AIDS [PLWHA]), a community-based Directly Observed Treatment (DOT) programme was established through a network of community volunteers. This programme demonstrated that community-level case holding was feasible. In 2005, ART was introduced in the rural hospital ("Hospital Rural de Ulongue") and CHWs were engaged to support adherence to ART by PLWHA. The rural hospital is a 78-bed, secondary-level hospital that provides basic medical and surgical care for adults and children. Only one medical doctor was working in the hospital at the time ART was introduced.

As of the beginning of 2007 there were five main types of CHW in Angónia. These groups, and their main activities, are described below (Table 1).

**Table 1:** Main results achieved through the different Community Health Worker (CHW) strategies

Strategy	Indicators	Before implementation	During implementation
▪ TB volunteers	▪ Number of suspected TB cases referred by volunteers	---	▪ 396 (2006)
			▪ 295 (2007)
	▪ % that reach the Health centre	---	▪ 68% (2006)
			▪ 72% (2007)
	▪ % of TB patients followed at community level	▪ 0% (2004)	▪ 67% (2006)
			▪ 67% (2007)
	▪ Defaulter rate	▪ 0% (2004)	▪ 0% (2006)
	▪ Completion rate (all new cases)	▪ 87% (2004)	▪ 74% (2006)
	▪ Cure rate (all BK+ new cases)	▪ 82% (2004)	▪ 91% (2006)
			
▪ APEs	▪ Number of consultations done by an APE	---	▪ 36,855 (2006)
▪ ACSs	▪ Vitamin A coverage	▪ 40.3% (2006)	▪ 60.8% (2007)
▪ TBAs	▪ % of institutional births	---	▪ 43.4% (2007)
	▪ Number of births attended by TBAs	---	▪ 459 (2007)
▪ HIV support groups (SG)	▪ Number of community SGs	---	▪ 35 (2007)
	▪ Number of members	---	▪ 270 (2007)
	▪ ARV defaulter rate in general	---	▪ 9% (06/2008)

#### Agente Polivalente Elementar (APE)

In the 1970s, basic health workers, "Agente Polivalente Elementar" (APE) were recruited from within the community and received six months of training to provide basic preventive (education) and curative health care at the community level. They were provided with a monthly supply of medications such as anti-malarial drugs, analgesics, antifungal ointments, antiseptics and dressings for wound care (for first aid). These drugs were sold to patients in the community (following the prevailing cost-recovery policy) and this provided a small income for CHWs. As of the end of 2008, eight APEs are still active in Angónia. The APEs allow isolated populations to have access to basic health care and essential drugs for high burden diseases like malaria and act as an entry point for referral to higher level health services, providing basic first aid before transfer where needed. However, most of the APEs abandoned their preventive role as this did not provide any financial compensation and was under-appreciated by the community.

#### TB volunteers

Between 2005 and 2006, one hundred volunteers from existing community groups were recruited as TB volunteers and given a two-day training and detailed task description (82 were still working in the district in 2008). Their activities included educating the community about TB, referral of TB suspects to the health centres, administration and follow up of treatment in the community and defaulter tracing. Their work was supported through regular supervision and ongoing training given by MoH health workers with MSF support. Material assistance was provided for their work, but they received no financial support.

In addition to referring large numbers of TB suspects to health structures, these volunteers succeeded in promoting the follow-up of TB patients at the community level against the prevailing DOT paradigm. Before the TB-volunteer strategy was implemented the national TB protocol required daily treatment directly observed by health staff during the intensive phase of TB treatment. This meant that many TB patients living far away from a health structure had to be hospitalized, which was impractical both for patients and the health services. The TB volunteers allowed people to receive their treatment directly at home, over time decreasing the number of visits, from weekly to monthly, until self-administered treatment was established. Treatment outcomes for community-administered TB treatment were comparable to those at the health centre level, while defaulting was higher at health centre level [14]. However, TB volunteers were not capable of raising TB detection rates to a substantial level. There are various explanations for this, including: uneven distribution and limited district coverage as no transport was provided; health facilities were not prepared to increase TB diagnosis; and limited acceptance by the community which saw the volunteers' role as being too narrow and not serving the major health needs of the community such as malaria (which accounts for over two-thirds of all child hospital admissions), diarrhoea, and malnutrition.

#### Agente Comunitário de Saúde (ACS)

At the end of 2006, a national campaign to provide vitamin A coverage for children under five years of age was launched by the MoH. Community health agents or "Agente Comunitário de Saúde" (ACS) were trained by the MoH to distribute vitamin A and to screen for cases of malnutrition for referral to the health centre. These community health agents were provided with the basic materials required to carry out these activities as well as bicycles to increase coverage. As with the TB volunteers, no financial compensation was provided. Currently, all ten community health agents that were initially trained are active in Angónia. Because recruitment, training and follow up were overseen by the local MoH staff the ACS's are accepted as part of the health system. However, as with the TB volunteers, this vertical and isolated strategy did not address other major health needs of the population.

#### Traditional Birth Attendants (TBA)

Since 1991, the MoH with the help of NGOs trained traditional birth attendants to provide basic support for pregnant women, refer them to health facilities and attend deliveries where needed. Approximately 100 TBAs are currently active in all 18 localities of the district. TBAs receive sporadic supplies of preventive material such as gloves and gowns from the MoH. They do not receive any financial incentives. TBAs provide advice for pregnant women and can provide basic care in case of emergency deliveries. However, lack of ongoing training means the quality of their work is highly variable, with problems of hygiene and prevention of disease transmission identified as concerns. Moreover, some of the TBAs overstep their roles by systematically assisting all deliveries and even charging for their services. This is partly a consequence of the unregulated nature of their work: the link between the TBAs and the health system is irregular.

#### HIV support groups

In 2006, PLWHA support groups were created at health centre level to provide psychosocial support and to promote long-term adherence to care and treatment among patients. In 2007, the support groups expanded into the community; they began to meet regularly in the villages and develop their own strategies to reinforce adherence. At the end of 2007, 38 groups were active in the district, providing the following services for members: sharing of information and experiences during monthly meetings; obtaining ARVs for those who could not attend their consultations at health centre level; tracing of defaulters; and social support (through collective fields or gardening). These groups are highly motivated and function on an independent basis and receive only occasional support from counsellors on technical issues. However, while these groups provide essential support services for their own members, they do not address other major problems in the community (which also affect them) such as maternal-child health care or malaria diagnosis and treatment. Moreover, since they were not selected by the community, their work is not always appreciated by the community.

### Towards a new strategy

**Figure 1:**
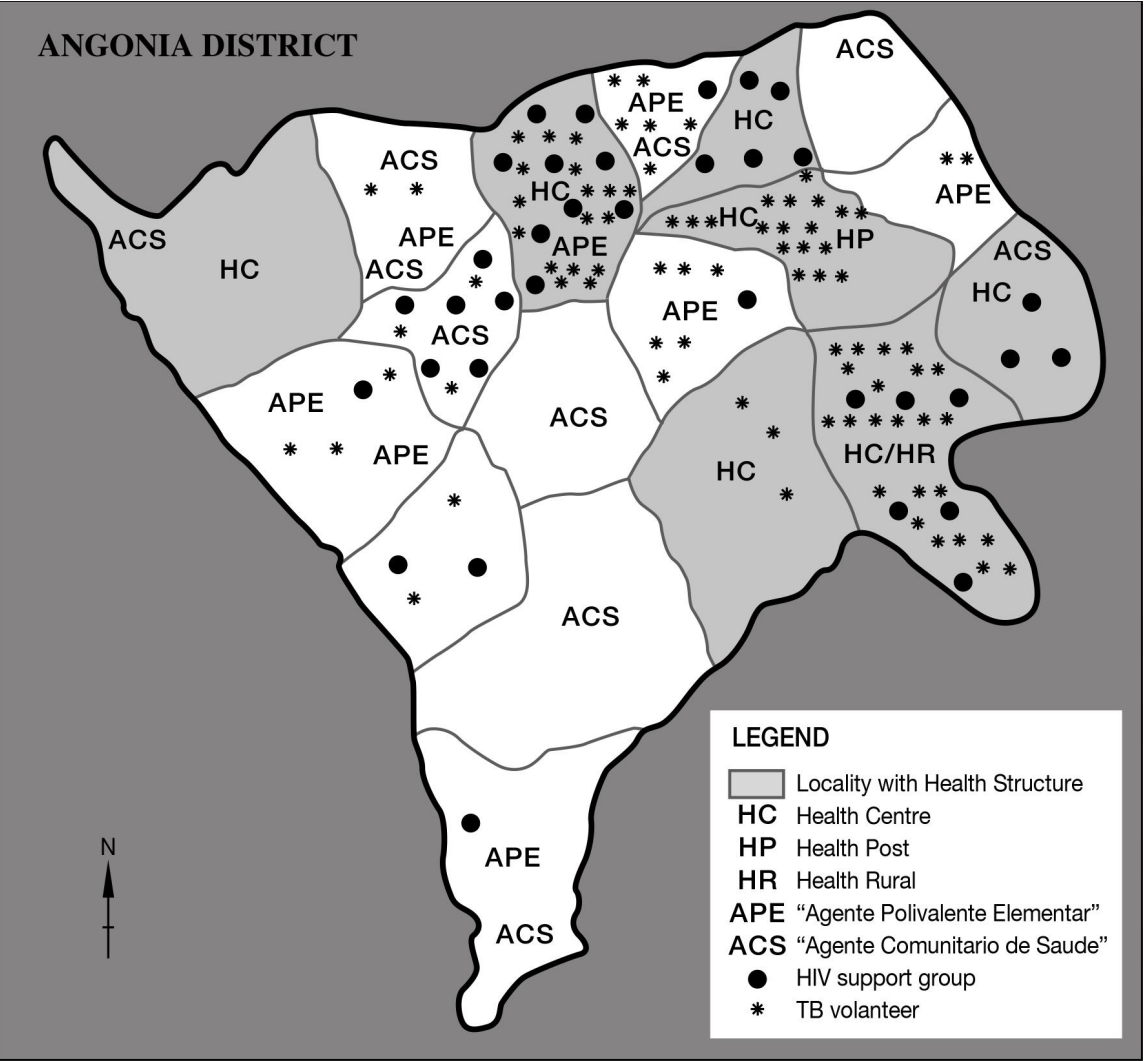
**Health worker coverage per locality in Angonia district (end 2007)**. NB TBAs not indicated as they are everywhere.

The results of these different approaches showed the potential of involving the community and patients in the management of health problems. However, these different types of community engagement evolved separately and functioned in isolation of each other: some worked with NGOs, others through the MoH, while others worked in complete isolation. The majority of the CHW groups had a vertical approach to health care, dealing with specific health problems. This inevitably meant that they were poorly perceived by some members of the community who felt that they were not attending to their priorities. Another issue was the heterogeneous coverage of their services: some areas had too many CHWs while others had none (Figure 1). Despite these limitations the potential of CHWs in supporting health care delivery was clear and this led to a move to engage CHWs in a more integrated manner.

### The community health team

In 2007, the MoH in Angónia together with MSF developed a strategy to maximise community participation in the management of a broad range of health problems via a community health team (CHT). This new approach aimed to provide coverage of the most basic health needs for the target area, bringing together different community actors and the formal health services (Table 2).

**Table 2:** Objectives and activities of the Community Health Team (CHT)

Objectives	Activities
▪ To provide health education for prevention and to improve health seeking behaviour for main diseases, TB, HIV.	▪ Information sessions for population organised with the local authority (all)
	▪ Information sessions for patients and their family (all)
▪ To detect and refer to the health centre suspected cases of TB, HIV related infections, malaria, diarrhoea, malnutrition.	▪ Visits to sick people (VCS)
	▪ Providing first aid when necessary (VCS, ACS)
	▪ Referral to health unit (VCS, ACS)
	▪ Organisation of transport when necessary (VCS, ACS)
	▪ Malnutrition screening (ACS)
	▪ Meeting at HC level (all)
▪ To support adherence for TB and HIV treatment.	▪ HIV testing (planned for 2009)
	▪ Distribution of TB treatment (VCS)
	▪ Community DOT (VCS)
	▪ Adherence support for patient and family (VCS)
	▪ Tracing of defaulters (all)
▪ To decrease stigma of PLWHA.	▪ Promotion of HIV test (all)
	▪ Information session (all)
▪ To enable family members to give basic care to their patient.	▪ Education offamily members (VCS)
▪ To increase institutional birth rate.	▪ Education session (TBA)
	▪ Referral of pregnant women (TBA)
▪ To increase Vitamin A coverage.	▪ Vitamin A distribution to children under 5 (ACS)

N.B. Category of CHW to whom each task is allocated is indicated in brackets.

The focal point of the CHT is the community health agent (ACS), who covers an area of around 15,000 people and acts as team leader for ten to fifteen community health volunteers or "Voluntario Comunitário de Saúde" (VCS). One VCS is chosen by the community for each "*chiwanga*" or group of villages; where possible they are chosen from the existing pool of CHWs (HIV support group member or former TB volunteer or APE). Other requirements include knowledge of the local language, willingness to collaborate with health staff, and the importance of maintaining confidentiality and respect.

Traditional birth attendants are also integrated in the CHT. As some VCS may also be members of HIV support groups, the team develops direct links with the local HIV support group in order to reinforce adherence and to facilitate defaulter tracing for TB and HIV. While the overall goal of the VCS is to integrate the previously disparate activities of the various CHWs, HIV support is maintained as a separate activity because of issues of confidentiality and the different function of the HIV support groups (peer support within the group rather than external support to others) (Table 2.)

Regular meetings with local authorities, community leaders, and the existing health volunteers are conducted to assess the main health problems identified by the community and to elaborate strategies to address these problems. The ACS and VCS in the team receive a five-day initial training. All members have regular bi-monthly meetings at health centre level to discuss difficult cases and to receive ongoing training and education. In order to facilitate the referral of patients or pregnant women from the community to health facilities, each community has been allocated two to three bicycle ambulances per locality under management of the members of the community.

CHT members receive no financial assistance. Rather, their activities are limited to just a few hours per week in order to allow them to engage in other work.

### Overview of achievements

Since the start of the CHT programme (November 2007), five teams are operational in five of the eighteen localities (December 2008). Preliminary results for these first two CHTs are shown in Table 3. While it is not possible to assess the impact in terms of increased coverage due to the absence of baseline data, the figures in terms of activities undertaken represent a substantial increase in services provided to the community. The fact that less than half of those referred to the health centre by the CHT actually reach the health centre was explained, in discussions with the VCS and health staff, by poor matching of data (not all referrals are recorded as such at the health centre) and the fact that some patients seek care at more proximal health centres across the border in Malawi.

**Table 3:** Main achievements of two of the CHTs

Indicators	Results
▪ Number of education sessions carried out by the CHT	▪ 464
▪ Number of people attending CHT education sessions	▪ 9,136
▪ Total number of referred patients by CHT/number who reach health centre:	▪ 1,130/231
- suspected malaria cases	- 260/45
- diarrhoea cases	- 58/26
- malnutrition cases	- 57/16
- TB cases	- 11/5
▪ Number of TB patients followed by CHT	▪ 15
▪ % of CHT members who participate in HC meetings	▪ 95%

## Discussion

The CHT has several advantages over the previous vertical CHW approaches.

First, a team approach ensures CHT members are accountable. Each member has to report to the team leader and to the rest of the team and the whole team has to report to the community and to the health centre about activities done and results obtained. Team members submit monthly reports to the health centre and these are discussed during meetings between the community health workers and the health centre nurses and included in the monthly district health report. This information is also fed back to the community via regular formal or informal meetings between the CHT members and community leaders.

Second, as a team, the CHT members can discuss difficult cases together and work with community leaders to find solutions. Unlike the previously fragmented organisation of CHWs, the CHTs offer broad health care coverage by providing a range of health care services. The CHTs also reach areas that previously did not benefit from CHW support. The CHT approach facilitates information flow between the community and the health facility and provides better coverage for activities such as adherence support and defaulter tracing of patients on ART or TB treatment.

Third, the results of the different experiences of CHW engagement, and of the CHT in particular, demonstrate the capacity of the community to improve geographic access to preventive and curative basic health services as described elsewhere [3]. The model has helped to overcome problems of distance and health service coverage faced by the formal health system through timely referral, additional transportation strategies (bicycle ambulances), and the decentralization of basic care.

For CHWs to be effective, good referral networks with the formal health system are required [3,4,16,17]. The CHT is not a substitute for the health system; rather, it acts as a complement by contributing to disease prevention activities, encouraging early health-seeking behaviour and providing basic care for acute conditions. Primary care nurses need to have the opportunity to work closely with the CHT to support routine vaccination and antenatal care consultations in the community, and should be the medical focal point for the CHT. For this to be successful, these nurses will need also to be properly trained and supported.

The previous diversity of community health actors in the same community created confusion for the population and led to an incoherence of messages and interventions and sometimes even conflict over responsibilities and approaches. Nevertheless, the needs are important and the different actors can be complementary if they are brought together, as the CHT experience has shown.

The CHW cadre of volunteers is critical in a setting like Angónia where the current health system lacks the human resources and infrastructure to serve a disparate population. Emerging public health issues such as the increase of multi-drug resistant TB and HIV make community health education and treatment adherence support imperative. Moreover, the CHT also recently demonstrated capacity to respond to emergencies by providing education about the dilution of chlorine in drinking water and the use of ORS during a cholera outbreak in 2009.

The MoH, as the responsible body for health delivery and coordination of all health partners, should take over the leadership to coordinate and unify the different actors of such an intervention. At the same time, the Angónia experience shows that NGOs have an important contribution to make by providing sufficient technical and financial support to help define innovative approaches to care provision. In the long term, motivation of the CHT members will need to be secured. The MoH is currently assessing a number of incentives, including the provision of free care for the members of the team and immediate family, the provision of bicycles to team members (for personal and professional use), and ensuring ongoing training and recognition. Such measures are essential for the long-term sustainability of a model that has proven invaluable to supporting health care in this underserved community.

## Competing interests

The authors declare that they have no competing interests.

## Authors' contributions

SS oversaw the design, implementation and follow-up of the programme and wrote the first draft of the article; MF, BC and MB participated in the definition and follow-up of the programme and were involved in writing the article; KC, NF and HS provided technical assistance for the writing of the article.

## Pre-publication history

The pre-publication history for this paper can be accessed here:

http://www.biomedcentral.com/1472-698X/9/13/prepub
